# Training Clinicians in Private Practice in Family-Based Treatment for Anorexia Nervosa: Randomized Controlled Trial Comparing Two Online Approaches

**DOI:** 10.2196/89999

**Published:** 2026-05-25

**Authors:** James Lock, Daniel Le Grange, Brittany Matheson, Bohye Kim, Cara Bohon, Booil Jo

**Affiliations:** 1 Department of Psychiatry and Behavioral Sciences, Stanford University School of Medicine, 401 Quarry Road, Stanford, CA, 94305, United States, 1 650-723-5511; 2 Department of Psychiatry and Behavioral Sciences, University of California, San Francisco (emeritus), San Francisco, CA, United States; 3 Department of Psychiatry and Behavioral Neuroscience, University of Chicago (emeritus), Chicago, IL, United States

**Keywords:** family-based treatment, anorexia nervosa, adolescents, implementation, evidence-based treatment, online training

## Abstract

**Background:**

There is a critical need to disseminate efficacious psychosocial treatments for mental disorders because there is a significant gap between evidence-based treatment (EBT) approaches and usual clinical practice. To address this gap, cost-effective, efficient, and scalable methods of training mental health clinicians in EBT are needed. One example of the need to improve dissemination and implementation of psychosocial treatments is for adolescents with anorexia nervosa (AN), a serious mental disorder with an incidence rate of about 1% that can become life-threatening. Based on outcomes from a series of randomized controlled trials (RCTs), an EBT for adolescents with AN is family-based treatment (FBT); however, few therapists are trained in the approach. Some studies suggest that online training is feasible for training clinicians treating eating disorders, including pilot data related to FBT specifically, but RCTs examining different training formats for FBT to improve fidelity and patient outcomes are needed.

**Objective:**

This study compared 2 different formats for delivering online training in FBT to therapists treating adolescents with AN and planned to assess the feasibility of the 2 training formats, as well as to examine whether either approach was superior in improving fidelity to FBT or patient outcomes.

**Methods:**

Participants were 123 mental health therapists licensed in the United States in private practice with no previous FBT training. Therapists were randomized to either (1) a webinar training lecture series or (2) an interactive e-training on-demand program (ET-FBT) with an additional focus on key FBT interventions. Both groups were offered up to 12 one-hour group-based clinical case consultation (CCC) sessions posttraining. We examined the feasibility and acceptability of the online training programs and posttraining outcomes in fidelity to key components of FBT, self-efficacy, and working alliance. We explored rates of patient early response (weight gain of 2.4 kg at session 4) in adolescent patients with AN treated after FBT training during CCC.

**Results:**

Both online trainings had a high completion rate of 95% (117/123), with CCC completion at 38% (47/123). Both programs showed significant improvements within randomized groups in therapists’ fidelity, self-efficacy, and working alliance. Working alliance improved significantly more in the ET-FBT group, but there were no other significant differences between training groups. Early response rates doubled posttraining and CCC (16%-34%), regardless of randomized training format, a rate that is similar to that achieved by therapists in RCTs examining FBT treatment outcomes.

**Conclusions:**

It is feasible to deliver online training in FBT to clinicians in private practice in 2 different formats, and both trainings led to significant improvements in fidelity, self-efficacy, working alliance, and patient outcomes. Future studies should address challenges in patient recruitment for posttraining CCC and refine CCC implementation to maximize training effects and efficiency.

## Introduction

There is a critical need to disseminate efficacious psychosocial treatments for mental disorders as there is a significant gap between evidence-based treatment (EBT) approaches and usual clinical practice [1-4]. One factor contributing to this gap is the lack of trained therapists [2,5]. The usual method for training therapists is in personal training workshops followed by supervision from experts, an approach that is both labor-intensive and costly [3]. One strategy to address these problems is the use of online training to more efficiently scale up training [6]. In a recent review of web-based training for mental disorders, including treatments for depression, anxiety, trauma, and eating disorders, the authors concluded that web-based training improved self-reported fidelity and outcomes, but additional studies were needed as training approaches were highly heterogeneous [7].

One area where there is a significant training need is for therapists treating eating disorders. Some studies suggest that online training is feasible for training clinicians treating eating disorders [8,9]. For example, Brownlow et al [10] found that an online training program for clinicians treating eating disorders improved knowledge and skills among participants. More recently, D’Adamo et al [8] examined the effects of online guided self-help cognitive behavioral therapy (CBT) for eating disorders and found the training platform and content to be feasible and acceptable to clinicians and families. Another study explored online training for interpersonal psychotherapy for eating disorders in a case series of 60 clinicians and found that the training was feasible, acceptable, and led to clinical improvements in patients [9]. No scaled randomized controlled trials (RCTs) have been conducted to examine online training for eating disorders.

One specific example of the need to improve the dissemination and implementation of psychosocial treatments is adolescents with anorexia nervosa (AN), a serious mental disorder with an incidence rate of about 1% [11-14] that can become life-threatening [15-17]. Based on outcomes from a series of RCTs, an EBT for adolescents with AN is FBT; however, few therapists are trained in the use of FBT for AN [18-20]. A significant amount of treatment for adolescents with AN is provided by clinicians in private practice, but most are not trained in EBT, and many patients do not receive EBT of any kind [21]. Motivations, incentives, and rationale for learning EBTs differ between therapists embedded in an organization or health care system and those in private practice [22-26]. Among the most salient differences is the personal financial impact of training costs and time allocation for clinicians in private practice rather than this being an institutional or programmatic burden. Nonetheless, clinicians in private practice are expected to follow clinical guidelines and adhere to professional community standards of practice, and they are, therefore, motivated to learn EBT when these treatments are desired by their clients, paid for by insurers, enhance their reputation as experts, and meet continuing education requirements [27].

Before embarking on an RCT examining online training for FBT-AN, we initially conducted a pilot study comparing 2 forms of online FBT training [28]. This pilot study had two principal aims: (1) to evaluate the feasibility and acceptability of randomization and the tolerability of study procedures by clinicians undergoing online training in FBT for adolescents with AN, and (2) to compare the relative efficacy of improving therapist competence in externalization and agnosticism in FBT (key components of FBT interventions) using 2 versions of online training (standard training FBT or enhanced training FBT [ET-FBT]). Standard training included training across all interventions of FBT, whereas the enhanced training provided additional emphasis by including short videos on externalization and agnosticism as 2 key components in FBT. To assess therapeutic competence, therapists were asked to complete an assessment focused on agnosticism and externalization. These key components are interventions used in FBT-AN that focus on reducing guilt and self-blame in parents by emphasizing that the cause of AN is unknown (ie, agnosticism) and separating the symptoms of AN from the identity and developmental issues of adolescents (ie, externalization) [29,30]. The results of this pilot study of the online trainings suggested that both trainings were generally acceptable, but attrition was 50% at the end of training (EOT), few participated in posttraining clinical case consultation (CCC), and only 10% of patient clinical outcomes were obtained [28]. There were few differences between the groups, except that the ET group showed greater improvements related to externalization and agnosticism skills, as expected [28]. Furthermore, the online training appears to have led to improvements specifically related to greater skill in some key components of FBT [28].

In this report, we describe the outcomes of a study comparing 2 forms of online training programs for FBT for adolescents with AN (FBT-AN) offered to therapists in private practice in the United States, followed by 12 hours of group clinical case consultation (CCC). Studies suggest that adding CCC posttraining improves treatment fidelity and clinical outcomes [31]. We predicted the following hypotheses based on the findings from our pilot study: (1) a 50% retention rate of clinicians in private practice in the study by the EOT, and (2) a 50% of those who started CCC would be retained by the end of follow-up. Our secondary hypotheses were that improvements in fidelity, self-efficacy, working alliance, and the percentage of patients achieving early weight gain (2.4 kg by session 4—a surrogate marker of end of treatment outcome for adolescents with AN treated with FBT) [32-34] and completing at least 4 CCC sessions (corresponding to session 4 of FBT)—would be greater in those randomized to ET-FBT than in clinicians who received webinar training (WT).

## Methods

### Study Design

The pilot study described above informed the design of this study as follows: (1) training clinicians in private practice—clinicians who treat a high proportion of youth with eating disorders but were not well represented in the pilot study and often do not receive training in EBTs, (2) refining materials on externalization and agnosticism in 1 training arm (ET-FBT) to confirm whether the preliminary data suggested that fidelity would improve with additional training in these key components of FBT, (3) incorporating strategies to improve retention by adding continuing medical education (CME) incentives (especially important for those in private practice who do not have institutional support for training), and (4) improving methods for capturing patient weight outcomes using therapist-deidentified reports because obtaining patient consent to collect these data, as attempted in the pilot study, was not feasible (too few patients consented) or effective (insufficient data were collected on outcomes to evaluate the impact of fidelity). This online training study was a longitudinal study with 3 primary assessment time points (baseline, end of online training, and end of CCC). A detailed description of the study protocol has been previously published [35].

### Setting

This study was conducted entirely virtually with no physical site locations. Private practice clinicians from across the United States were invited to participate. Recruitment and data collection occurred from November 2020 to October 2024.

### Participants

Therapists were required to be licensed (Masters, PhD, PsyD, and MD [psychiatrist]) in the United States and confirm that they regularly treated adolescents with AN in the context of a private practice and that they had not previously been trained in FBT. Methods of recruitment included—social media posts, professional organizations, and listservs (Twitter [subsequently rebranded X], Academy of Eating Disorders, Eating Disorder Referral and Information Center, and licensing organizations). Interested therapists were directed to an online questionnaire for further study information and screened for eligibility. Prior to online training, eligible therapists reported weight change from sessions 1 to 4 of an adolescent with AN they had recently treated using any treatment other than FBT. This weight change would be compared to weight change by session 4 of FBT post–online training during CCC to explore whether training affected early weight gain. Baseline assessments were conducted prior to training allocation and were completed online. After completing assessments, therapists were randomized to either ET-FBT or WT using block randomization. The training period for each group lasted approximately 3 months and was followed by 3 months of weekly CCC.

### Assessments

Detailed descriptions of the measures and related procedures used in this study can be found in a previously published protocol paper related to this study’s design [35]. Therapists completed the measures detailed below at baseline, at the end of online training, and at the end of CCC. In addition, the consultants who conducted CCC completed the *Supervisor Report of Fidelity to Key Components of FBT* at the end of 12 hours of CCC [29,30]. Consultants were faculty members at one of the participating sites in this study. Both consultants were trained in FBT and certified by the Training Institute for Child and Adolescent Eating Disorders as consultant supervisors. The consultants were masked to the participant training condition.

The list of measures used in this study is as follows:

- Demographic and professional survey: participants answered questions related to demographic characteristics, including professional licensure status, highest degree attainment, years in private practice, and years working in their current position.
- The therapist version of the Parents Versus Anorexia Nervosa Scale [36]: this 7-item self-report measure is adapted from the Parents Versus Anorexia Nervosa Scale [36] to assess therapists’ self-efficacy with regard to their knowledge and skills in FBT.
- Evidence-Based Practice Attitude Scale–36 [37]: this 36-item measure is a brief version of the Evidence-Based Practice Attitude Scale–50 that assesses clinicians’ attitudes toward evidence-based practice, with demonstrated psychometric validity and reliability [37]. This measure was administered at baseline only.
- Motivation for Training in FBT Questionnaire: this measure was created by the research team to assess therapists’ motivations for pursuing FBT training. This questionnaire asks participants to select and rank from the following reasons: belief that FBT is the best approach, customer demand, curiosity about FBT, increased income, and professional development. This measure was administered at baseline only.
- Key Component Knowledge Questionnaire [28]: this 10-item multiple choice measure assesses knowledge about the key components of FBT. This measure was previously used in the pilot online training trial to assess the applied knowledge of the key components of FBT by study participants [28].
- Fidelity to FBT Self-Report to Key Components: this is an 8-item multiple choice measure used to assess self-reported fidelity to key components of FBT delivered in the first 4 sessions of treatment. It has been piloted and found acceptable by FBT therapists.
- Working Alliance Inventory (WAI) [38]: the WAI is a 12-item self-report assessment of the therapeutic working alliance with well-established psychometric properties. Participants were asked to complete the measure as it related to the patient for whom posttraining weight data was provided.
- Patient weight change: weight gain from sessions 1 to 4, as reported by the therapist for the patient treated during CCC (and only that patient, if more than 1 patient was discussed), was used as the patient outcome (early weight gain of 2.4 kg by session 4*).*

### Study Size

The sample size for this study was determined to facilitate our investigation focusing on improvements in therapist outcomes, including self-efficacy, working alliance, and fidelity. These outcomes were not primary in this study, although they were critical for planning and enriching the next step of the confirmatory efficacy trial. We estimated power based on the proposed linear mixed effects (LME) modeling with repeatedly measured outcomes (baseline [pretraining], end-of-training [EOT], and follow-up [end of CCC]). We assumed a modest intraclass correlation of 0.5 for the repeated measures, with 50% attrition by follow-up. We also assumed a medium effect size (Cohen *d*=0.4-0.5) as a clinically meaningful minimum effect size. Under this scenario, with the expected combined sample of 140 clinicians at pretraining, the estimated power to detect significant improvement in therapist competency from baseline to follow-up is 0.98‐1.0 (2-tailed; α=.05). In terms of retention, our primary outcome, we set at least 50% retention by EOT and at least 50% retention by follow-up among those who start CCC as a success. Assuming about 65% retention at EOT, using a 1-sample chi-square test, the estimated power to achieve retention significantly better than 50% is 0.95. For our exploratory investigation, including group comparisons (ET-FBT vs WT) in therapist and patient outcomes and detecting baseline moderators of group effects, we planned to monitor both clinical significance (ie, effect sizes) and statistical significance (*P* value), with more emphasis on identifying clinical significance.

### Training Descriptions

The 2 online training strategies, as noted in the “Study Size” section, are described more fully in a study by Citron et al [35], but a brief description here provides a summary of the main features of the 2 trainings. The WT arm includes an online webinar lecture series. In this arm, participants watched a weekly video lecture (approximately 1 h) created by authors JL and DLG, based on the *Treatment Manual for Anorexia Nervosa: A Family-Based Approach* [39]. Participants had 1 week to view the lecture, and the materials were not available for review. The lectures discuss the scientific evidence supporting FBT, how therapists set up treatment for FBT, the main interventions used in FBT during each phase, and recorded role-plays illustrating interventions throughout the 3 phases. The Enhanced Training (ET-FBT) arm uses an online training platform that releases 1 of 7 lecture bundles every 12 days. Once released, participants had access to the training material throughout their participation in the study. The ET-FBT lecture bundles consisted of 5 to 8 short didactic videos that discuss the treatment model and provide mock therapy session video clips (modeling FBT with a typical adolescent with AN case), supplementary readings (such as the *Treatment Manual for Anorexia Nervosa: A Family-Based* Approach [39]), clinical vignettes and session roleplays, and training assignments. Participant assignments included questions on the medical management of adolescent patients with AN, reflection on clinical vignettes and the therapist’s approach, and identification of treatment moderators. ET-FBT included additional focused training related to the key components of FBT—agnosticism and externalization. Brief online didactic lectures defined the 2 principles and discussed their uses, challenges, and application in clinical practice. Participants were provided with clinical vignettes on the application of agnosticism and externalization. Both trainings lasted approximately 12 total hours.

### Ethical Considerations

Eligible therapists were consented using a protocol approved by the institutional review board at Stanford University and the University of California, San Francisco (e-protocol 56548). All participants gave their informed consent for inclusion before participating in the study. The authors assert that all procedures contributing to this work comply with the ethical standards of the relevant national and institutional committees on human experimentation and with the 1975 Declaration of Helsinki, as revised in 2008. Therapists did not pay for training, but to enhance retention, they received CME credit for completing the training and CCC. Participants were compensated US $100 for each assessment time point (US $300 total). All reports are private and confidential per the institutional review board–approved protocols.

### Data Analytic Plan

To estimate improvement in clinician outcomes, we used linear mixed effects (LME) modeling [40,41] with random intercepts and maximum likelihood estimation, which enabled us to fully use outcome data repeatedly measured for most clinicians (3 assessment time points). Given the 2 distinct phases in our study design (baseline to EOT and EOT or initiation of CCC to completion of CCC), we used piecewise growth models that allowed different slopes from baseline to EOT and from EOT to follow-up. We used the training group (ET-FBT vs WT) as the predictor of changes across the 2 phases. The piecewise LME model we used here is presented below.

We describe our general model for longitudinal LME modeling in 2 levels. The outcome *Y* for therapist *i* at time point *t* can be expressed as follows:


(1) $$Y_{it}=\eta_{0i}+\eta_{1i}S_{1t}+\eta_{2i}S_{2t}+\varepsilon_{i,}$$

which is a function of 3 growth parameters:


(2) $$\eta_{0i}=\alpha_{0}+\gamma_{0}Z_{i}+\zeta_{0i},$$

(3) $$\eta_{1i}=\alpha_{1}+\gamma_{1}Z_{i},$$

(4) $$\eta_{2i}=\alpha_{2}+\gamma_{2}Z_{i},$$

where *i*=1, 2, . . . , *N* (*N*=123 in our study) and *t*=0, 1, 2 (baseline, EOT, and follow-up). Therapists were randomly assigned to 1 of the 2 conditions (*Z*_*i*_=1 if ET-FBT, *Z_i_*=0 if WT). We modeled the longitudinal trend using 3 growth parameters, the initial status (*η*_0_*_i_*) and the changes from the baseline to EOT (*η*_1_*_i_*) and from EOT to follow-up (*η*_2_*_i_*). We used time scores of *S*_1_*_t_* (=0, 1, 1) for the first and *S*_2_*_t_* (=0, 0, 1) for the second slope. The fixed effects of interest are the mean initial status (*α*_0_ under WT, *α*_0_+*γ*_0_ under ET-FBT), the changes in each treatment group (*α*_1_, *α*_2_ under WT, *α*_1_+*γ*_1_ , *α*_2_+*γ*_2_ under ET-FBT) and the group differences in these changes (*γ*_1_and *γ*_2_). We allow for individual variability at baseline (ie, random intercept), which is captured by the variance of level 2 residual (Σ_*ζ*_=Var(*ζ*_0_*_i_*)). We assumed the random effect to be normally distributed (*ζ*_0*i*_~N(0,Σ_*ζ*_)). We used the standard assumption for the level 1 residual, which is normally distributed and consistent across assessments (*ε_i_*~*N*(0,σ^2^)).

In our LME modeling, data points that were missing due to clinician attrition or intermittent dropout were handled under the assumption that data are missing at random [42] conditional on observed information, which is a standard assumption in modern missing data approaches. In this procedure, all available cases, including those with missing information, were included in the analysis. For maximum likelihood estimation of mixed effect models, we used the *lme4* package [42] in R software (R version 4.4.1; R Foundation for Statistical Computing). For the exploratory patient outcome, early weight gain by session 4 (restoring 2.4 kg or not), we planned to report simple proportions at baseline and follow-up as the information is not collected repeatedly from the same individuals. We also explored the number of years working in private practice and therapists’ age at baseline as possible treatment effect moderators and/or predictors of therapist outcomes and patient early weight response.

## Results

### Demographics of Clinicians Trained

Participants were approximately 10% Hispanic, 9% non-White, and 96% female, with a mean age of 36.9 (SD 8.8) years (Table 1). Most were master’s level clinicians (77%), followed by psychologists (23%) and psychiatrists (1%). Recruitment and data collection occurred from November 2020 to October 2024.

**Table 1.** Participant characteristics (N=123).

Characteristics	ET-FBT^a^ (n=61)	WT^b^ (n=62)	Total (N=123)
Ethnicity, n (%)			
Hispanic	7 (11.5)	5 (8.1)	12 (9.8)
Non-Hispanic	54 (88.5)	57 (91.9)	111 (90.2)
Sex, n (%)			
Male	3 (4.9)	1 (1.6)	4 (3.3)
Female	58 (95.1)	61 (98.4)	119 (96.7)
Race, n (%)			
American Indian or Alaskan Native	0 (0)	0 (0)	0 (0)
Asian	1 (1.6)	1 (1.6)	2 (1.6)
Black or African American	0 (0)	1 (1.6)	1 (0.8)
Native Hawaiian or other Pacific Islander	0 (0)	0 (0)	0 (0)
White	55 (90.2)	57 (91.9)	112 (91.1)
More than 1 race	5 (8.2)	3 (4.8)	8 (6.5)
Age (y), mean (SD)	36.4 (7.2)	37.5 (10.1)	36.9 (8.8)
Licensure, n (%)			
Master’s	48 (78.7)	47 (75.8)	95 (77.2)
Psychiatrist or MD	0 (0)	1 (1.6)	1 (0.8)
Psychologist (PhD, PsyD)	12 (19.7)	14 (22.6)	26 (21.1)
Education specialist	1 (1.6)	0 (0)	1 (0.8)
Years of private practice experience, mean (SD)	4.1 (4.2)	4.6 (6.0)	4.3 (5.2)

a ET-FBT: enhanced training family-based treatment.

b WT: webinar training.

### Recruitment, Feasibility, and Acceptability

**Figure 1.**
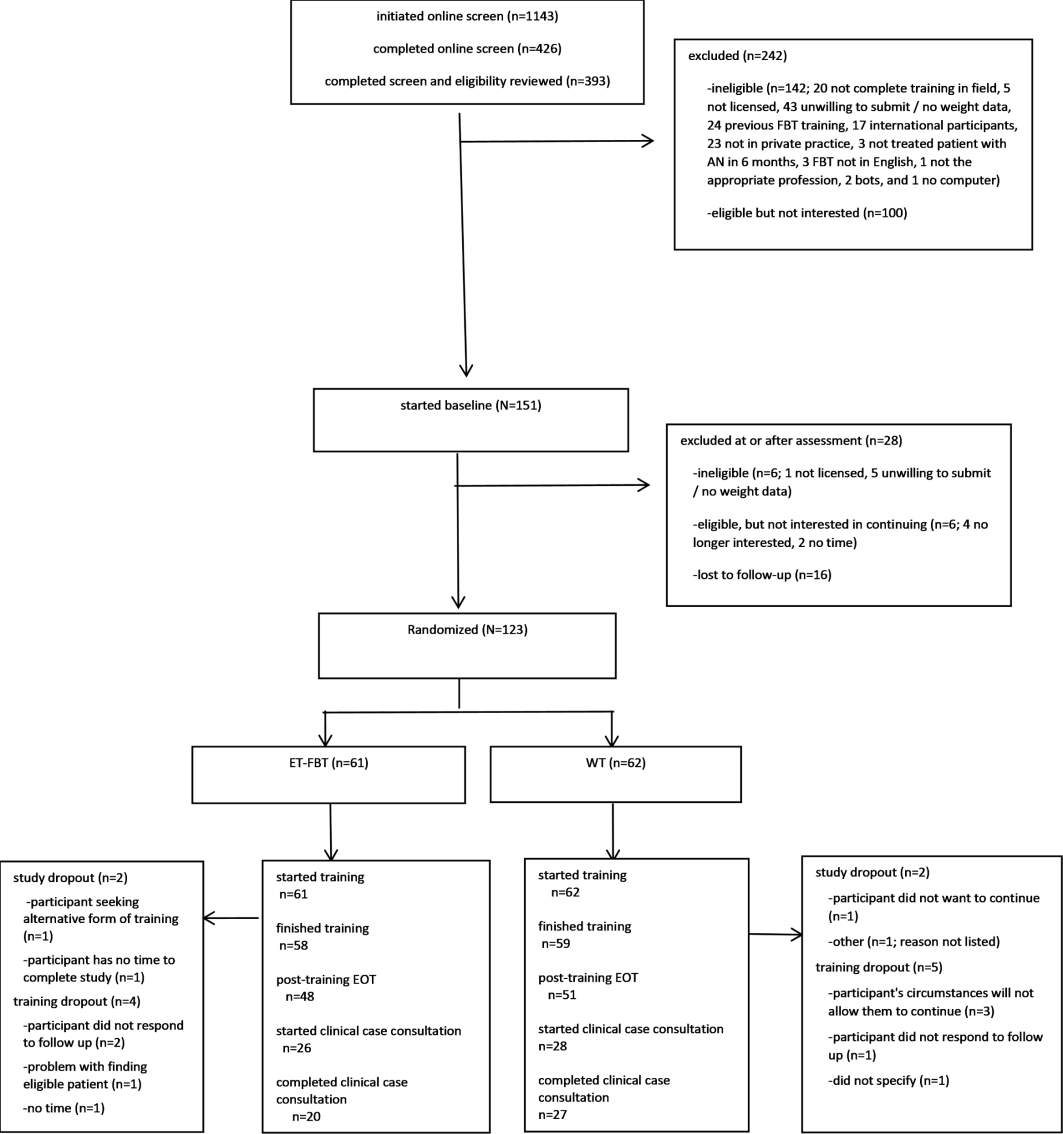
CONSORT (Consolidated Standards of Reporting Trials) diagram. AN: anorexia nervosa; EOT: end-of-training; ET-FBT: enhanced training family-based treatment; FBT: family-based treatment; WT: webinar training.

We randomized 123 patients (88% of the expected number; Figure 1) over the course of 3 years. Participants were recruited from 33 states, with the largest percentage practicing in California (n=26, 21%), Virginia (n=11, 9%), New York (n=8, 6.5%), North Carolina (n=8, 6.5%), Michigan (n=8, 6.5%), Texas (n=7, 5.7%), and Florida (n=7, 5.7%). A total of 20 states (Alabama, Alaska, Arkansas, Colorado, Connecticut, Idaho, Indiana, Iowa, Maryland, Massachusetts, Minnesota, Missouri, Montana, Nevada, Ohio, Oregon, Rhode Island, South Carolina, Washington, and Wisconsin) had 3 or fewer participants. A total of 123 clinicians started training, and 117 (95%) completed it. Of those who completed online training, 89 (72%) planned to start CCC, but only 54 (44%) found an eligible patient and started CCC during the allotted timeline for study completion.

### Training Outcomes

There was evidence of considerable improvement in the training outcome measures within both treatment arms (Table 2). In TVAN and fidelity self-ratings, both arms showed similarly large effect sizes (Cohen *d* of 1.0-1.9) in terms of changes from baseline to EOT and follow-up (end of CCC), leading to insignificant group differences. In WAI, both groups showed similar modest but significant improvements from baseline to EOT. However, by follow-up, the ET-FBT condition showed significantly more improvement (Cohen *d*=0.53; *P*=.04) in working alliance (WAI). Expert fidelity ratings at the end of CCC, provided by the consultants who were blind to the training condition, did not suggest a difference between the groups (WT=21.3 (SD 4.21) and ET-FBT=19.3 (SD 6.93); Cohen *d*=−0.35, 95% CI −0.94 to 0.24; *P*=.25). These expert ratings of fidelity were somewhat lower than self-reported fidelity but were significantly correlated with each other (*r*=0.33; *P*=.03).

**Table 2.** Estimated changes and intention to treat effects based on longitudinal mixed effects.

	Within group change										Group difference in change (ET-FBT vs WT)		
	ET-FBT^a^					WT^b^							
	Mean (95% CI)		Change (95% CI)	ES^c^	*P* value	Mean (95% CI)		Change (95% CI)	ES	*P* value	Difference in change (95% CI)	ES	*P* value
TVAN^d^													
Baseline to EOT^e^	Baseline: 23.21 (22.42 to 24.01)	EOT: 28.26 (27.39 to 29.13)	5.05 (4.19 to 5.91)	1.49	<.001	Baseline: 23.42 (22.63 to 24.21)	EOT: 28.01 (27.17 to 28.85)	4.59 (3.76 to 5.42)	1.36	<.001	0.46 (−0.74 to 1.66)	0.14	.45
Baseline to follow-up	Baseline: 23.21 (22.42 to 24.01)	Follow-up: 29.68 (28.51 to 30.86)	6.47 (5.30 to 7.64)	1.90	<.001	Baseline: 23.42 (22.63 to 24.21)	Follow-up: 29.33 (28.24 to 30.43)	5.92 (4.83 to 7.00)	1.75	<.001	0.55 (−1.04 to 2.15)	0.16	.50
EOT to follow-up	EOT: 28.26 (27.39 to 29.13)	Follow-up: 29.68 (28.51 to 30.86)	1.42 (0.23 to 2.60)	0.42	.02	EOT: 28.01 (27.17 to 28.85)	Follow-up: 29.33 (28.24 to 30.43)	1.32 (0.23 to 2.42)	0.39	.02	0.09 (−1.52 to 1.71)	0.03	.91
WAI^f^													
Baseline to EOT	Baseline: 58.81 (56.47 to 61.14)	EOT: 63.59 (61 to 66.18)	4.78 (2.00 to 7.56)	0.44	.001	Baseline: 56.9 (54.59 to 59.22)	EOT: 60.12 (57.62 to 62.62)	3.22 (0.52 to 5.91)	0.36	.02	1.56 (−2.31 to 5.44)	0.16	.43
Baseline to follow-up	Baseline: 58.81 (56.47 to 61.14)	Follow-up: 67.18 (63.57 to 70.78)	8.37 (4.62 to 12.11)	0.76	<.001	Baseline: 56.9 (54.59 to 59.22)	Follow-up: 59.95 (56.61 to 63.29)	3.05 (−0.44 to 6.54)	0.34	.09	5.32 (0.19 to 10.44)	0.53	.04
EOT to follow-up	EOT: 63.59 (61.00 to 66.18)	Follow-up: 67.18 (63.57 to 70.78)	3.59 (−0.23 to 7.40)	0.33	.07	EOT: 60.12 (57.62 to 62.62)	Follow-up: 59.95 (56.61 to 63.29)	−0.17 (−3.71 to 3.38)	−0.02	.93	3.75 (−1.46 to 8.96)	0.37	.16
Fidelity self-rating													
Baseline to EOT	Baseline: 10.67 (9.1 to 12.25)	EOT: 17.45 (15.72 to 19.17)	6.77 (5.06 to 8.48)	1.03	<.001	Baseline: 10.39 (8.83 to 11.95)	EOT: 16.21 (14.53 to 17.89)	5.82 (4.16 to 7.49)	0.96	<.001	0.95 (−1.44 to 3.34)	0.15	.47
Baseline to follow-up	Baseline: 10.67 (9.1 to 12.25)	Follow-up: 21.02 (18.69 to 23.35)	10.35 (8.03 to 12.66)	1.58	<.001	Baseline: 10.39 (8.83 to 11.95)	Follow-up: 21.42 (19.26 to 23.59)	11.03 (8.88 to 13.19)	1.82	<.001	−0.69 (−3.85 to 2.48)	−0.11	.67
EOT to follow-up	EOT: 17.45 (15.72 to 19.17)	Follow-up: 21.02 (18.69 to 23.35)	3.58 (1.23 to 5.93)	0.55	.003	EOT: 16.21 (14.53 to 17.89)	Follow-up: 21.42 (19.26 to 23.59)	5.21 (3.02 to 7.41)	0.86	<.001	−1.64 (−4.85 to 1.58)	−0.26	.32

a ET-FBT: enhanced training family-based treatment.

b WT: webinar training.

c ES: effect size (in Cohen *d*)*.*

d TVAN: Therapist Versus Anorexia Nervosa Scale.

e EOT: end-of-training.

f WAI: Working Alliance Inventory.

### Patient Outcomes

The primary patient-related outcome for this study was early weight gain of 2.4 kg by session 4, used as a proxy predictor (surrogate marker) for end-of-treatment weight restoration [32-34]. The percentage of early weight gain of 2.4 kg by session 4 for the sample at baseline (prior to online training) was 15.4% (19/123). At follow-up, the percentage of patients who achieved early weight gain of 2.4 kg by session 4 more than doubled in both training approaches to a rate of 34% (33% under ET-FBT; 35% under WT) compared to the rate at baseline. This rate of early response is almost identical to that achieved by trained and supervised FBT therapists participating in RCTs [43].

### Potential Moderators and Predictors of Therapist and Patient Outcomes

We examined the number of years working in private practice and therapists’ age at baseline as possible treatment effect moderators and/or predictors of self-reported fidelity. The number of years working in private practice did not show a significant association with self-reported fidelity either at EOT or follow-up. Therapist age showed little or negative association with self-reported fidelity in the WT arm (EOT: *r*=0.13, *P*=.36; follow-up: *r*=–0.39, *P*=.048), whereas it showed a positive association with fidelity in the ET-FBT arm (EOT: *r*=0.32, *P*=.03; follow-up: *r*=0.26, *P*=.25). As a result, at follow-up, the moderating effect of age was significant (*r* difference=0.65; *P*=.04), which can be interpreted as older therapists benefiting more from the ET-FBT condition. We did not find any significant associations between baseline therapist characteristics and patient early weight response.

## Discussion

### Principal Findings

The results of this study found that both online training strategies were acceptable and feasible to conduct with clinicians in private practice. They led to improvements in self and expert reported fidelity to the key components, self-efficacy related to the delivery of FBT, and working alliance. Furthermore, both training strategies also appeared to lead to improved patient outcomes, more than doubling the early response rates compared to the rates of those treated prior to FBT training. This is a rate that approximates what is achieved in RCTs with expert FBT clinicians [43]. The only difference between the two training approaches was that the WAI showed significantly greater increases in the ET-FBT group, which may be attributable to baseline differences in this measure. Thus, overall, our hypothesis that ET-FBT would outperform WT was not supported. While this result was not as expected, it is important that both training programs led to fidelity to FBT key components and patient clinical improvements. This supports the promise that online training of therapists in FBT is likely a viable way to increase the supply of therapists who can deliver the treatment with reasonable fidelity, with an expectation that their patients will experience substantial benefit. These results are in line with previous nonrandomized studies of online training for clinicians treating eating disorders, where significant improvements in skills and confidence were noted for clinicians learning CBT or interpersonal psychotherapy [8,9]. Taken together, these studies support the conclusion that online training is likely a feasible and practical strategy for training clinicians in the main evidence-based approaches for eating disorders.

The recruitment process was hampered in part by the COVID-19 pandemic, resulting in only 123 participants (88% of our original aim of 140) enrolling. This was because many therapists reported being overwhelmed by referrals and therefore had no time for training [44-46]. Nonetheless, interest in receiving online training in FBT was high, with 1143 expressing interest, 426 completing the online screening, 251 being deemed eligible after screening, 123 starting the training, and 117 completing it (95%), which was much higher than the 50% we had anticipated based on our pilot studies. However, in this study, of those who completed the online training, 89 (72%) expressed interest in receiving CCC, but only 54 (44%) of these clinicians were able to find an eligible patient and start CCC within the allotted timeline for study completion. This was lower than the 50% we had anticipated. At the same time, the majority of those who found an eligible patient and started CCC completed the 12 hours offered (n=47, 87%) and provided patient outcome data (ie, patient weight change from sessions 1‐4). This level of obtaining patient outcome data was much greater than the 10% we were able to collect in the pilot study, where obtaining patient consent was a major barrier [28]. Overall, the acceptability of study procedures was high, with rates of completion of study measures at the EOT reaching 79% (48/61) for ET-FBT and 82% (51/62) for WT, and with an overall rate of assessment completion of 80% (99/123), with no statistical differences between randomized groups.

As noted above, it was somewhat surprising and contrary to our hypothesis that additional training in agnosticism and externalization did not improve fidelity. We speculate that, while the didactic content in the ET-FBT group may have improved intellectual understanding of these interventions, participating in CCC provided additional learned application of these skills in a clinical case, which leveled up fidelity in both groups. Alternatively, it may be that the preliminary studies overestimated the benefits of these additional training modules and that standard training is already sufficient for clinicians’ understanding and use of agnosticism and externalization.

While the EOT results were comparable between groups, the predicted attrition of 50% posttraining participation in CCC was evident in this trial. However, a large proportion of those who did not complete CCC were unable to start (38%) because they could not find an eligible patient in time. As suggested above, this may be a result of the fact that much of the study took place during COVID-19, when there was a significant uptick in eating disorders, and therapists treating eating disorders were overwhelmed with referrals and did not have the time or energy for training. However, a more important factor might have been the study eligibility requirement that patients treated in the study be underweight at the start of FBT, which precluded the enrollment of adolescents who were partially weight-restored in other settings or who had atypical AN without low weight. Many clinicians in private practice reported seeing mostly patients who were weight-restored in other settings (hospitals, day programs, and residential programs). Of those who did complete CCC, there was an early weight response by session 4 of FBT after only 4 hours of CCC. At the same time, those who completed all 12 hours of CCC saw continued improvement in fidelity and self-efficacy (TVAN) in both training arms, as well as WAI in the ET-FBT arm (Table 2).

Because early weight response is a strong predictor of end-of-treatment weight restoration, these data provide support that training plus CCC led to clinical implementation effects on patient outcomes similar to those expected in RCTs. The early weight response did not differ by treatment group (odds ratio 0.99, 95% CI 0.67-1.47). However, the study design did not include the collection of patient outcome data beyond session 4 of FBT. This was because weight gain at this early time point is a reliable predictor of end-of-treatment remission, with a predictive value of 70% to 80% in RCTs [32], and also because the study’s funding duration was limited to a 3-year window. However, by not including non-low weight adolescents with AN in this sample, we cannot know for certain whether session 4 weight gain will be a predictor of end-of-treatment response for this clinical group, and future studies that include all adolescents with AN, regardless of starting weight, are needed to clarify this.

This study was designed using a practical approach to real-world challenges related to future training and implementation procedures, as well as clinical outcome measurement. As a result, we focused on clinician-reported data as a feasible approach to diagnostic assessment, fidelity, and patient weight outcomes. Therefore, the clinician-participants identified eligible patients in their practice (ie, diagnosed with AN both prior to and during the training period). The clinician participants also reported weight at baseline and session 4 for the identified patients they treated or would treat during the training period. These design features allowed for real-world evaluation of training and patient outcomes but also resulted in limitations regarding related findings resulting from self-report. It is possible that some clinicians inaccurately diagnosed or reported inaccurate weight change data. However, there was no evidence or any clear motivation for false reporting, and there were no differences in the changes between the 2 online training platforms, suggesting that if these inaccuracies were present, they were equally distributed between the randomized groups.

This study used a standard dose of CCC post training because studies suggest that CCC post training is a necessary aspect of mastering the skills described in a didactic training [31,47,48]. However, CCC is a challenge to scale because it is costly, and there are limited experts to provide it. A next step would be to systematically examine cost-effective strategies for CCC, including determining more specifically how much CCC is needed to ensure fidelity and clinical outcomes, as well as strategies for more efficient procedures of CCC, including potentially group approaches. This is an important area for future investigations. Furthermore, it is unknown if the additional 8 hours of CCC beyond the first 4 had any effect on patient outcomes. CCC post online training is likely necessary to implement EBT effectively [31], so future studies should identify how many hours of CCC are needed in addition to the online training to develop an evidence-based and scalable FBT training model combining these two essential elements.

Another limitation of this study design is the exclusive focus on private practice and majority White clinicians, thereby limiting generalization to clinicians of other ethnicities or races and those who practice in other settings. However, there is little reason to expect that clinicians in other clinical settings or even those outside the United States would not respond similarly, especially given our previous pilot study [28]. We used training incentives, specifically CME, to encourage clinicians in private practice to attend training. We did this because clinicians in private practice need CMEs for continued licensure and to help mitigate the loss of income related to training. Clinicians in public practice settings or those with organizational support may not need this type of incentive. Thus, for clinicians in these other settings, costs to provide training would likely be lower without the costs of CME included in the training.

By design, most of our outcome measures are self-reported, and additionally, some do not have psychometric studies documenting their reliability. However, this study used measures that were short and scalable so that they had viability in a real-world clinical context where the training of therapists and evaluation of fidelity must be efficient and practical. Thus, while the measures have only face validity, they appear to capture self-reported change on key variables related to fidelity and self-efficacy in the delivery of FBT. Importantly, the key measure of therapist self-reported fidelity was correlated with expert consultant fidelity ratings, suggesting that self-report may be a reasonable and practical strategy to assess fidelity in a clinical setting, but this requires additional study. In addition, future studies should determine the psychometric properties of these fidelity measures with greater precision.

Finally, although patients treated during CCC more than doubled the rates of early response (15% vs 34%) from pretraining to posttraining, supporting an important patient outcome change effect, there was no comparison group that did not receive CCC posttraining; therefore, it is not possible to determine if and how much additional CCC is needed to improve early weight gain.

### Conclusions

This study demonstrates that it is feasible, acceptable, and practical to train therapists in private practice in FBT-AN using 2 different online strategies, with little evidence that one has advantages over the other. It is important to note that the main reason participants were excluded from training was that they were not in private practice or could not find an eligible patient with baseline weight data, rather than due to a lack of interest, as 59% of eligible participants enrolled and most of those (95%) completed online training. In addition, the outcomes of the study suggest that there were improvements in all measured training and fidelity metrics, as well as patient early response rates. These data, taken together with our previous studies, suggest that asynchronous online FBT training could be offered to clinicians in private practice at low cost using either of the approaches tested in this study. These training materials can be distributed easily through learning platforms and translated into non-English versions using relatively inexpensive artificial intelligence translation technologies. However, several key questions remain: What are the benefits of adding CCC to online training, and how much CCC is needed to achieve fidelity to the key components of FBT and to achieve expected patient outcomes? Challenges in the uptake of CCC were evident in this trial, and additional strategies to increase uptake and completion of CCC are needed. Because CCC is potentially costly and the availability of expert consultants is limited, it is important to clarify for individual providers and health care systems an expected effective strategy. Making CCC as short as feasible and associated with a predictable outcome would likely encourage more therapists to take up and complete CCC.

This study portends a new era for FBT training availability, which should, in turn, lead to an increase in trained and competent providers, as well as improved access for young persons with AN and their families to an important evidence-based intervention for this potentially lethal disorder.

## Supplementary material

10.2196/89999 Checklist 1 CONSORT checklist.

## Abbreviations

AN: anorexia nervosa
CCC: clinical case consultation
CME: continuing medical education
EBT: evidence-based treatment
EOT: end of training
ET: enhanced training
FBT: family-based treatment
LME: linear mixed effects
RCT: randomized controlled trial
WAI: working alliance inventory
WT: webinar training
